# Causal effects of the gut microbiome on COVID-19 susceptibility and severity: a two-sample Mendelian randomization study

**DOI:** 10.3389/fimmu.2023.1173974

**Published:** 2023-09-01

**Authors:** Meng-Mei Zhong, Jia-Hao Xie, Yao Feng, Shao-Hui Zhang, Jiang-Nan Xia, Li Tan, Ning-Xin Chen, Xiao-Lin Su, Qian Zhang, Yun-Zhi Feng, Yue Guo

**Affiliations:** ^1^ Department of Stomatology, The Second Xiangya Hospital, Central South University, Changsha, Hunan, China; ^2^ Institute of Artificial Intelligence & Robotics (IAIR), Key Laboratory of Traffic Safety on Track of Ministry of Education, School of Traffic and Transportation Engineering, Central South University, Changsha, Hunan, China; ^3^ Department of Stomatology, Xiangyang Central Hospital, Xiangyang, Hubei, China; ^4^ School of Architecture and Art, Central South University, Changsha, Hunan, China

**Keywords:** gut microbiome, Mendelian randomization, COVID-19, susceptibility, severity

## Abstract

**Background:**

The coronavirus disease 2019 (COVID-19) caused a global pandemic, with potential severity. We aimed to investigate whether genetically predicted gut microbiome is associated with susceptibility and severity of COVID-19 risk.

**Methods:**

Mendelian randomization (MR) analysis of two sets with different significance thresholds was carried out to infer the causal relationship between the gut microbiome and COVID-19. SNPs associated with the composition of the gut microbiome (n = 5,717,754) and with COVID-19 susceptibility (n = 14,328,058), COVID-19 severity (n = 11,707,239), and COVID-19 hospitalization (n = 12,018,444) from publicly available genome-wide association studies (GWAS). The random-effect inverse variance weighted (IVW) method was used to determine causality. Three more MR techniques—MR Egger, weighted median, and weighted mode—and a thorough sensitivity analysis were also used to confirm the findings.

**Results:**

IVW showed that 18 known microbial taxa were causally associated with COVID-19. Among them, six microbial taxa were causally associated with COVID-19 susceptibility; seven microbial taxa were causally associated with COVID-19 severity ; five microbial taxa were causally associated with COVID-19 hospitalization. Sensitivity analyses showed no evidence of pleiotropy or heterogeneity. Then, the predicted 37 species of the gut microbiome deserve further study.

**Conclusion:**

This study found that some microbial taxa were protective factors or risky factors for COVID-19, which may provide helpful biomarkers for asymptomatic diagnosis and potential therapeutic targets for COVID-19.

## 1 Introduction

The coronavirus disease 2019 (COVID-19) is an infectious disease of potential severity, with more than 200 symptoms identified and effects on multiple-organ systems (1). According to the World Health Organization (WHO), as of 23 July 2023, over 768 million confirmed cases and over 6.9 million deaths have been reported globally (2). This is due to the highly contagious nature of the respiratory-transmitted virus and the fact that SARS-CoV-2 has evolved variants (e.g., α, β, γ, δ, and Omicron) that may escape from neutralizing antibodies and/or cell-mediated immunity, resulting in general susceptibility to the population (3, 4). The virus mainly affects the respiratory system, but studies have found that several patients (27.3%) had gastrointestinal symptoms but no COVID-19 lung imaging findings, indicating that gastrointestinal tissues are vulnerable to SARS-CoV-2 (5). Moreover, the gastrointestinal tract is the largest immune organ in humans and plays a key role in the host’s fight against pathogenic infection (6). Trillions of microbiota colonize the human gut and are involved in host immunomodulation (7). Therefore, it is important to understand the relationship between the gut microbiome and the susceptibility and severity of SARS CoV-2 to the host, the changes in the gut microbiome after host infection with SARS CoV-2, and the long-term impact on human health (including long-term COVID-19 syndrome, “LongCOVID”).

Recently, research on the correlation between the gut microbiome and COVID-19 has attracted widespread attention. There is evidence that the gut microbiome influences the susceptibility and severity of COVID-19. On the one hand, the recruitment of immune cells to the lung may be adversely affected by gut microbiome disruption, which may therefore raise the susceptibility to respiratory infections (8, 9). On the other hand, COVID-19 has been linked to changes in the gut microbiome and impaired gut barrier function, which may enhance the translocation of bacterial products and toxins into the circulatory system and aggravate the systemic inflammatory response (10, 11). Additionally, some studies have shown that the fecal microbiome of COVID-19 patients shows reduced richness and diversity of the gut microbiome (12, 13). Although most current clinical studies suggest that the gut microbiome may be associated with the pathogenesis and disease outcome of COVID-19, most studies are observational epidemiology and have limitations such as confounding factors and causal inversion (14). Thus, the causal associations between gut microbiome and COVID-19 outcomes remain unconfirmed.

Genetic tools, often single-nucleotide polymorphisms (SNPs), are used in Mendelian randomization (MR) research to identify the causal relationships between exposures and outcomes. As either allele has an equal chance of being randomly inherited by a person, MR investigations are comparable with randomized controlled trials (15). MR investigations are more capable of drawing conclusions about causality and are less prone to confounding problems than observational studies like case–control studies (16). This study used two-sample MR to determine whether there is a causal relationship between the composition of the gut microbiome and the risk of COVID-19, as well as to look for pathogenic bacteria and probiotics for COVID-19.

## 2 Materials and methods

### 2.1 Genome-wide association study

**Figure 1:**
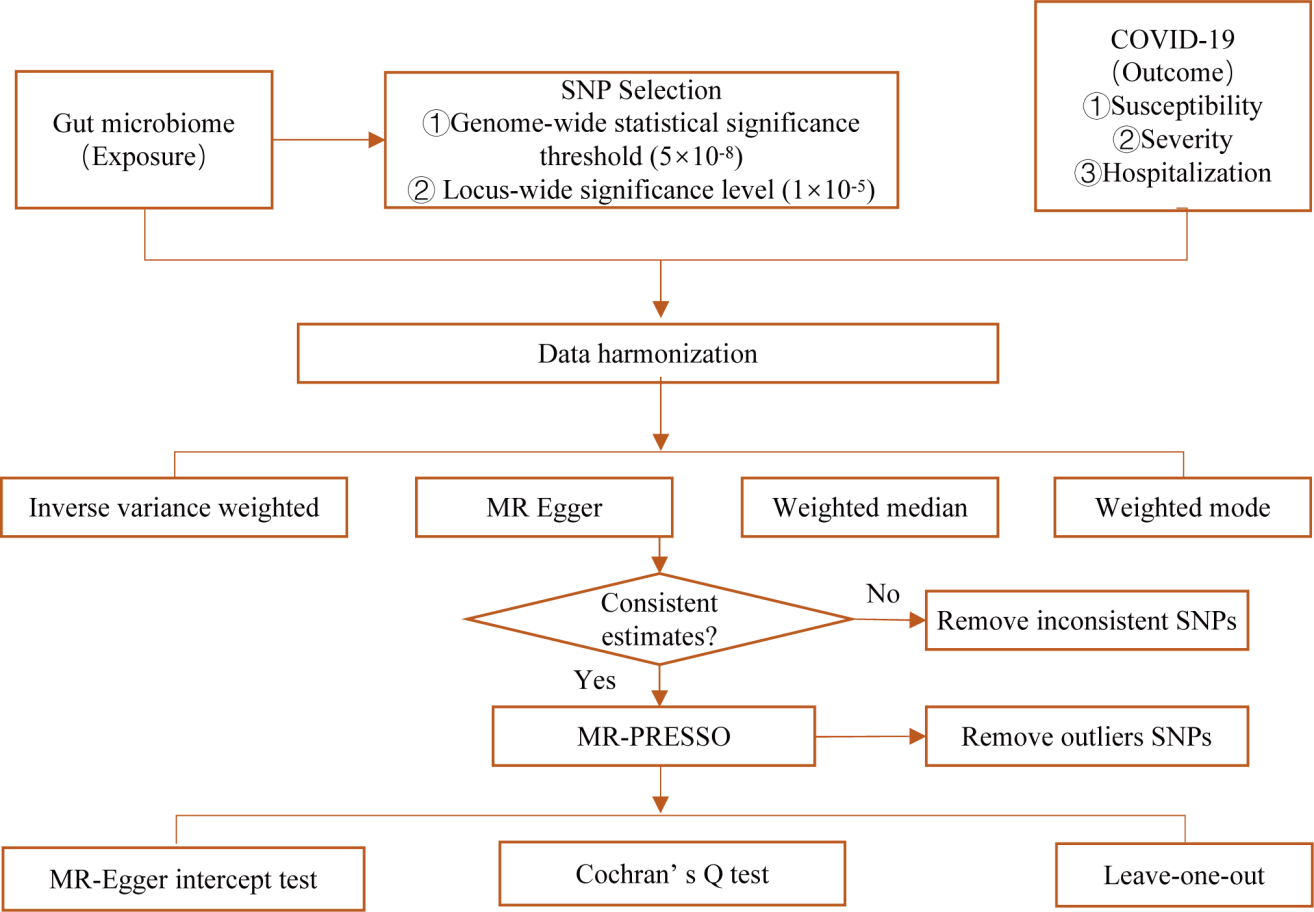
Diagrammatic explanation of the entire MR analysis procedure.

Summary-level statistics of gut microbiome and COVID-19 (including susceptibility, severity, and hospitalization of COVID-19) for genome-wide association study (GWAS) were obtained from the public database. All studies were approved by their respective institutional review boards (IRBs). No new IRB approval was required. A flowchart briefly presents the whole procedure in **Figure 1**.

Genetic instruments of the gut microbiome were obtained from 18,340 participants in 24 cohorts, which is the largest, multiethnic, genome-wide meta-analysis of the gut microbiome to date (17) (**Table 1**). For each cohort, quantitative microbiome characteristic locus (mbQTL) profiling included only taxa in >10% of the sample, containing 211 taxa (9 phyla, 16 classes, 20 orders, 35 families, and 131 genera). Aggregate statistics on the Association’s research are publicly available at https://mibiogen.gcc.rug.nl/.

**Table 1:** Description of the GWAS summary statistics.

Trait	Sample size (case)	Number of SNPs	Population	References
Gut microbiome	18,340	5,717,754	European	PMID: 33462485
COVID-19 susceptibility (COVID-19 *vs*. population)	159,840 *vs*. 2,782,977	14,328,058	Mixed	PMID: 32404885
COVID-19 severity (very severe respiratory confirmed *vs*. population)	18,152 *vs.* 1,145,546	11,707,239	Mixed	PMID: 32404885
COVID-19 hospitalization (hospitalized *vs*. population)	44,986 *vs.* 2,356,386	12,018,444	Mixed	PMID: 32404885

Summary-level statistics of COVID-19 susceptibility, severity, and hospitalization for the selected instrument variables were obtained from the COVID-19 Host Genetics Initiative (18) (**Table 1**). GWAS on COVID-19 susceptibility involved 159,840 patients with COVID-19 and 2,782,977 population controls, whereas GWAS on COVID-19 severity involved 18,152 very severe respiratory confirmed patients with COVID-19 and 1,145,546 population controls. Meanwhile, we also analyzed GWAS on COVID-19 hospitalization which involved 44,986 hospitalized patients with COVID-19 and 2,356,386 population controls. Aggregate statistics on the Association’s research are publicly available at https://www.covid19hg.org/, Release 7.

### 2.2 Selection of instrumental variables

The following quality control procedures were utilized to choose the best instrument factors to assure the validity and correctness of the results about the causal relationship between gut microbiome and COVID-19 risk. SNPs with a strong correlation to the gut microbiome were first chosen as the instrumental factors. To choose the instrumental variable, two thresholds were applied. The instrumental variables were a group of SNPs that did not meet the genome-wide statistical significance cutoff ( $5\times 10^{-8}$ ). The other group, where SNPs are less than the locus-wide significance threshold ( $1\times 10^{-5}$ ), was chosen as an instrumental factor to acquire more thorough results. The linkage disequilibrium (LD) threshold was set to be $r^{2}<0.001$ , with a clumping window of 10,000 kb. To guarantee a high connection between IVs and exposure variables, the F-statistic of each SNP was utilized to assess the correlation strength and prevent bias brought on by weak IVs. Weak IVs were thought to be bias-free when the F value was more than 10. An important step in MR is ensuring that SNPs’ effect on exposure corresponds to the same allele as the effect on the outcome. According to this principle, palindromic SNPs are removed.

### 2.3 Mendelian randomization analysis

We hypothesized that the gut microbiome influences the risk of COVID-19 and satisfied the assumption that SNP is an instrumental variable correlated with gut microbiome levels; SNP is not associated with any confounding factors; SNP is associated with COVID-19 via gut microbiome (i.e., horizontal pleiotropy should not be present).

We used the two-sample MR analysis utilizing the random-effect inverse variance weighted (IVW) approach, which is the most popular in MR research and could offer reliable causal estimates in the absence of directional pleiotropy, to assess the impact of the gut microbiome on COVID-19 (19). MR Egger, weighted median, and weighted mode were also used to further validate the causal results (20, 21). Also, Mendelian Randomization Pleiotropy RESidual Sum and Outlier (MR-PRESSO) analysis was performed to find outlier instruments and then removed inconsistent SNPs (22). Finally, extensive sensitivity studies were needed to assess any model assumption breaches in the MR study. The Cochran’s Q test was performed to determine if there is heterogeneity among the various causal effects, and MR–Egger intercept analysis was used to assess the horizontal pleiotropy (23). Moreover, leave-one-out analysis was performed to assess the effect of individual variation on the observed association, which refers to eliminating each SNP step by step, calculating the meta-effect of the remaining SNPs, and observing whether the result changes after removing each SNP.

### 2.4 Clinical exploration

In this MR study, the gut microbiome database’s lowest taxonomic level was genus. However, we need to identify the species of the gut microbiome if we want to explore the mechanism between the gut microbiome and disease development. Therefore, according to the results of the genus, we searched the species included in the genus using the UniProt Knowledgebase (UniProtKB) (24) and determined whether the species belongs to the human gut microbiome through GMrepo (data repository for Gut Microbiota) (25).

### 2.5 Statistical analysis

All analyses were performed using R software (version 4.2.1) under the Windows environment. The R packages used for all MR-related analysis and image plotting included “TwoSampleMR” and “MR-PRESSO”. Based on the IVW method, a two-sided p<0.05 was considered statistically significant. Additionally, we used the 0.05 threshold for the FDR correction (*p*-FDR). When p<0.05 and p−FDR<0.05 , the causal connection was considered significant. In addition, when p<0.05 but p−FDR>0.05 , the causal connection was considered potentially significant.

## 3 Results

### 3.1 At the genome-wide statistical significance threshold (5×10^-8^), there is no causal relationship between gut microbiome and COVID-19 risk

With the genome-wide statistical significance threshold of $5\times 10^{-8}$ , MR analysis was performed with the gut microbiome as a whole, and a total of 36 SNPs were included in the study. For each SNP, the F-statistics varied from 29.81 to 85.38, indicating a low likelihood of weak instruments. In the IVW analysis, we observed evidence of causal associations of the gut microbiome without risks of COVID-19 susceptibility (odds ratio (*OR*) = 1.042; 95% confidence interval (*CI*) = 0.970–1.118; *p* = 0.259), and COVID-19 severity (*OR* = 1.071; 95% *CI* = 0.889–1.291; *p* = 0.469), and COVID-19 hospitalization (*OR* = 1.067; 95% *CI* = 0.943–1.207; *p* = 0.304). MR–Egger regression showed no horizontal pleiotropy between instrumental variables and outcome. IVW heterogeneity testing indicated heterogeneity ( p<0.05 ) but did not affect IVW results. The detailed description is summarized in **Table 2**.

**Table 2:** MR results of causal links between the gut microbiome and COVID-19 risk *p*<5×10^−8^.

	Nsnp	Method	Beta	SE	P value	OR (95% CI)	Horizontal pleiotropy			Heterogeneity	
							Egger intercept	SE	P value	Cochran’s Q	P value
COVID-19 susceptibility	12	MR–Egger	−0.060	0.127	0.644	0.941 (0.734–1.207)	0.011	0.014	0.424	81.605	7.21E−13
		Weighted median	0.034	0.020	0.089	1.035 (0.995–1.077)					
		Inverse variance weighted	0.041	0.036	0.259	1.042 (0.970–1.118)					
		Weighted mode	0.043	0.026	0.126	1.043 (0.992–1.097)					
COVID-19 severity	12	MR–Egger	−0.227	0.326	0.503	0.797 (0.420–1.511)	0.033	0.035	0.366	55.472	6.35E−08
		Weighted median	0.029	0.059	0.624	1.030 (0.916–1.157)					
		Inverse variance weighted	0.069	0.095	0.469	1.071 (0.889–1.291)					
		Weighted mode	0.016	0.073	0.827	1.016 (0.881–1.173)					
COVID-19 hospitalization	12	MR–Egger	−0.109	0.221	0.633	0.897 (0.581–1.384)	0.019	0.024	0.431	56.604	3.94E−08
		Weighted median	0.005	0.040	0.897	1.005 (0.929–1.088)					
		Inverse variance weighted	0.065	0.063	0.304	1.067 (0.943–1.207)					
		Weighted mode	0.004	0.044	0.931	1.004 (0.921–1.094)					

### 3.2 At the locus-wide significance level ( $1\times 10^{--5}$ ), a causal relationship was identified between gut microbiome and COVID-19 risk

**Figure 2:**
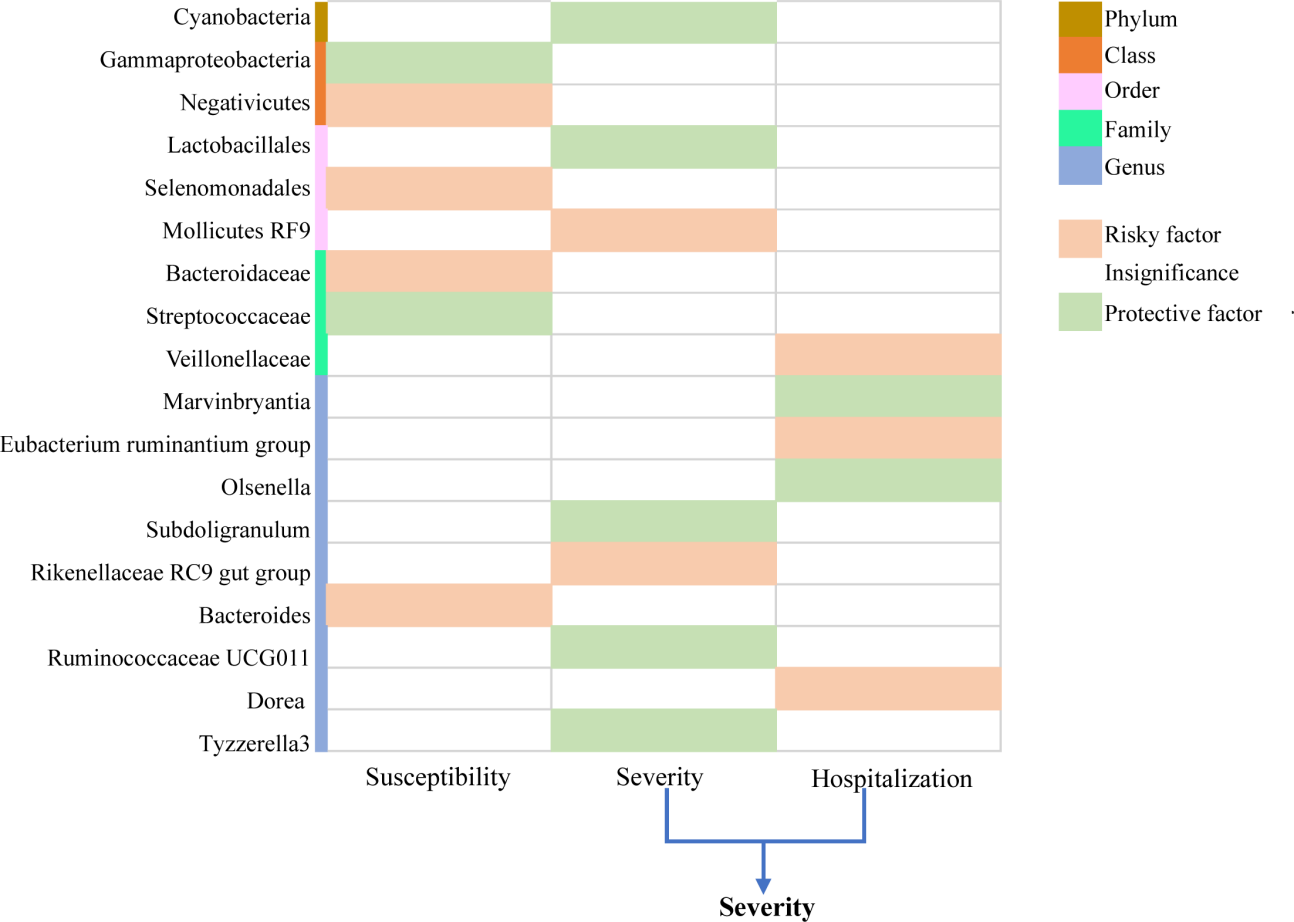
Causal relationship between gut microbiome and COVID-19 susceptibility, COVID-19 severity, and COVID-19 hospitalization at the locus-wide significance level ( $1\times 10^{-5}$ ).  p < 0.05 .

A total of 235 SNPs were identified as instrumental variables for predicted gut microbiome mass. For each SNP, the F-statistics varied from 17.68 to 35.42, indicating a low likelihood of weak instruments. Furthermore, we observed some significant evidence for the causal association between gut microbiome and COVID-19 risk, based on IVW methods. Finally, 18 known named microbial taxa were identified at a significance of 0.05, including one phylum, two classes, three orders, three families, and nine genera (**Figure 2**). Thereinto, two microbial taxa were protective factors and four microbial taxa were risky factors of COVID-19 susceptibility; seven microbial taxa were protective factors, and five microbial taxa were risky factors of COVID-19 severity and hospitalization. The detailed description is summarized in **Supplementary Tables 1**–**3**. However, after FDR adjustment, the aforementioned correlations were no longer statistically significant ( p−FDR>0.05 ). This suggests that the above microbial taxa have potential causal suggestions for COVID-19.

### 3.3 MR effect of the gut microbiome on COVID−19 susceptibility

**Figure 3:**
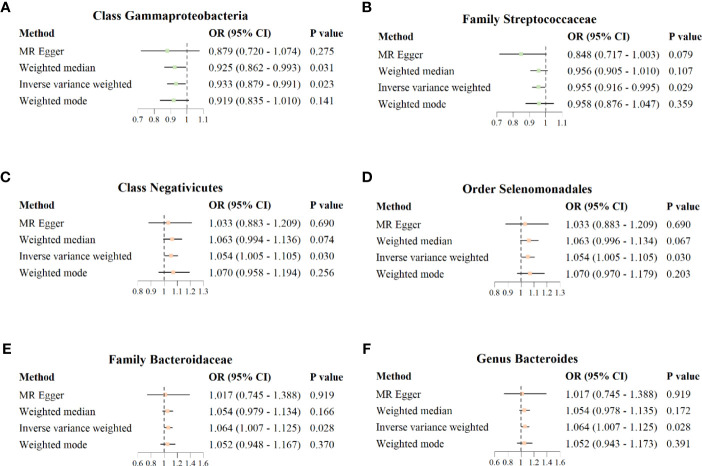
Forest plot for causal association between six unique protective/risky microbial taxa with COVID-19 susceptibility. **(A, B)** Two protective microbial taxa for COVID-19 susceptibility. **(C–F)** Four risky microbial taxa for COVID-19 susceptibility.

The results of IVW analysis showed that class *Gammaproteobacteria* (*OR* = 0.933; 95% *CI* = 0.879–0.991; *p* = 0.023) and family *Streptococcaceae* (*OR* = 0.955; 95% *CI* = 0.916–0.995; *p* = 0.029) were negatively correlated with the risk of COVID−19 susceptibility; class *Negativicutes* (*OR* = 1.054; 95%*CI* = 1.005–1.105; *p* = 0.030), order *Selenomonadales* (*OR* = 1.054; 95% *CI* = 1.005–1.105; *p* = 0.030), family *Bacteroidaceae* (*OR* = 1.064; 95% *CI* = 1.007–1.125; *p* = 0.028), and genus *Bacteroides* (*OR* = 1.064; 95% *CI* = 1.007–1.125; *p* = 0.028) were positively correlated with the risk of COVID−19 susceptibility. MR Egger, weighted median, and weighted mode also showed the same direction of influence as IVW, although the *P* value was not statistically significant (**Figure 3**).

### 3.4 MR effect of the gut microbiome on COVID−19 severity

**Figure 4:**
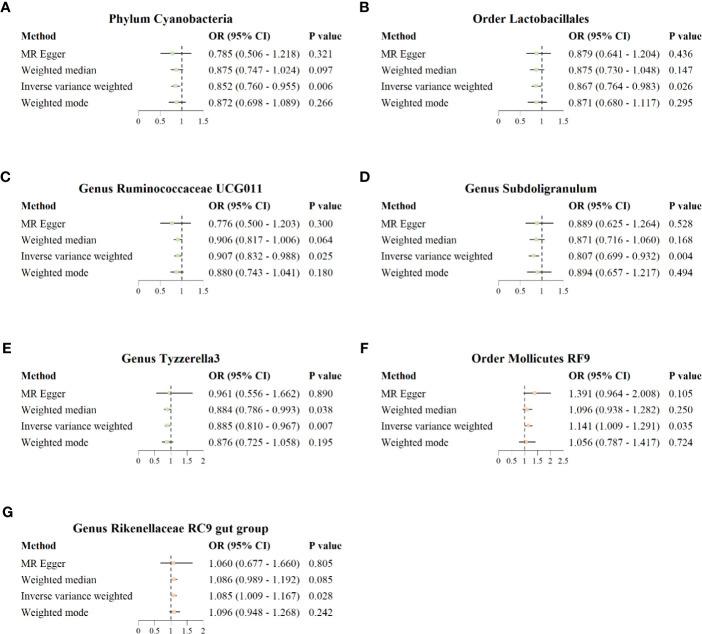
Forest plot for causal association between seven unique protective/risky microbial taxa with COVID-19 severity. **(A–E)** Five protective microbial taxa for COVID-19 severity. **(F, G)** Two risky microbial taxa for COVID-19 severity.

The results of IVW analysis showed that phylum *Cyanobacteria* (*OR* = 0.852; 95% *CI* = 0.760–0.955; *p* = 0.006), order *Lactobacillales* (*OR* = 0.867; 95% *CI* = 0.764–0.983; *p* = 0.026), genus *Ruminococcaceae UCG011* (*OR* = 0.907; 95% *CI* = 0.832–0.988; *p* = 0.025), genus *Subdoligranulum* (*OR* = 0.807; 95% *CI* = 0.699–0.932; *p* = 0.004), and genus *Tyzzerella3* (*OR* = 0.885; 95% *CI* = 0.810–0.967, *p* = 0.007) were negatively correlated with the risk of COVID−19 severity; order *Mollicutes RF9* (*OR* = 1.141; 95% *CI* = 1.009–1.291; *p* = 0.035) and genus *Rikenellaceae RC9 gut group* (*OR* = 1.085; 95% *CI* = 1.009–1.167; *p* = 0.028) were positively correlated with the risk of COVID−19 severity (**Figure 4**). In addition, the results of IVW analysis showed that genus *Marvinbryantia* (*OR* = 0.886; 95% *CI* = 0.812–0.967; *p* = 0.007) and genus *Olsenella* (*OR* = 0.942; 95% *CI* = 0.897–0.990; *p* = 0.018) were negatively correlated with the risk of COVID-19 hospitalization; family *Veillonellaceae* (*OR* = 1.069; 95% *CI* = 1.002–1.140; *p* = 0.044), genus *Eubacterium ruminantium group* (*OR* = 1.065; 95% *CI* = 1.010–1.123; *p* = 0.021), and genus *Dorea* (*OR* = 1.162; 95% *CI* = 1.055–1.279; *p* = 0.002) were positively correlated with the risk of COVID-19 hospitalization. MR–Egger, weighted median, and weighted mode also showed the same direction of influence as IVW, although the *P* value was not statistically significant (**Supplementary Figure 1**).

### 3.5 Sensitivity analysis

A comprehensive sensitivity analysis was then performed to assess the robustness of the causal relationship between the gut microbiome and COVID-19 above. Firstly, the results of the MR-PRESSO trial showed no horizontal pleiotropic outliers. Secondly, no evidence of a horizontal pleiotropic effect was observed using the MR–Egger intercept analysis. In addition, heterogeneity testing also indicated that there is no heterogeneity. The detailed description is summarized in **Table 3**. Furthermore, the leave-one-out analysis revealed that none of the included SNPs was outliers. The plots of the leave-one-out analysis are shown in **Supplementary Figure 2**.

**Table 3:** Heterogeneity and horizontal pleiotropy analyses results *p *<1×10^-5^.

Outcome			Nsnp	Horizontal pleiotropy			Heterogeneity	
				Egger intercept	SE	P value	Cochran’s Q	P value
COVID-19 susceptibility	Class	Gammaproteobacteria	6	0.005	0.007	0.574	0.955	0.966
	Class	Negativicutes	12	0.001	0.005	0.805	8.351	0.682
	Order	Selenomonadales	12	0.001	0.005	0.805	8.351	0.682
	Family	Bacteroidaceae	9	0.003	0.010	0.780	8.252	0.409
	Family	Streptococcaceae	14	0.009	0.007	0.179	8.977	0.775
	Genus	Bacteroides	9	0.003	0.010	0.780	8.252	0.409
COVID-19 severity	Phylum	Cyanobacteria	8	0.011	0.028	0.716	7.048	0.424
	Order	Lactobacillales	15	−0.001	0.012	0.929	9.472	0.800
	Order	Mollicutes RF9	13	−0.017	0.015	0.286	13.940	0.305
	Genus	*Rikenellaceae* RC9 gut group	11	0.003	0.032	0.919	3.743	0.958
	Genus	*Ruminococcaceae* UCG011	8	0.021	0.029	0.504	2.074	0.956
	Genus	*Subdoligranulum*	11	−0.008	0.013	0.570	6.363	0.784
	Genus	*Tyzzerella3*	13	−0.012	0.039	0.770	14.633	0.262
COVID-19 hospitalization	Family	Veillonellaceae	19	−0.001	0.005	0.847	17.742	0.473
	Genus	*Olsenella*	11	0.000	0.012	1.000	9.193	0.514
	Genus	*Marvinbryantia*	10	0.014	0.015	0.388	4.837	0.848
	Genus	*Dorea*	10	0.011	0.009	0.239	8.703	0.465
	Genus	*Eubacterium ruminantium* group	18	−0.008	0.009	0.407	15.044	0.592

### 3.6 Clinical exploration

**Figure 5:**
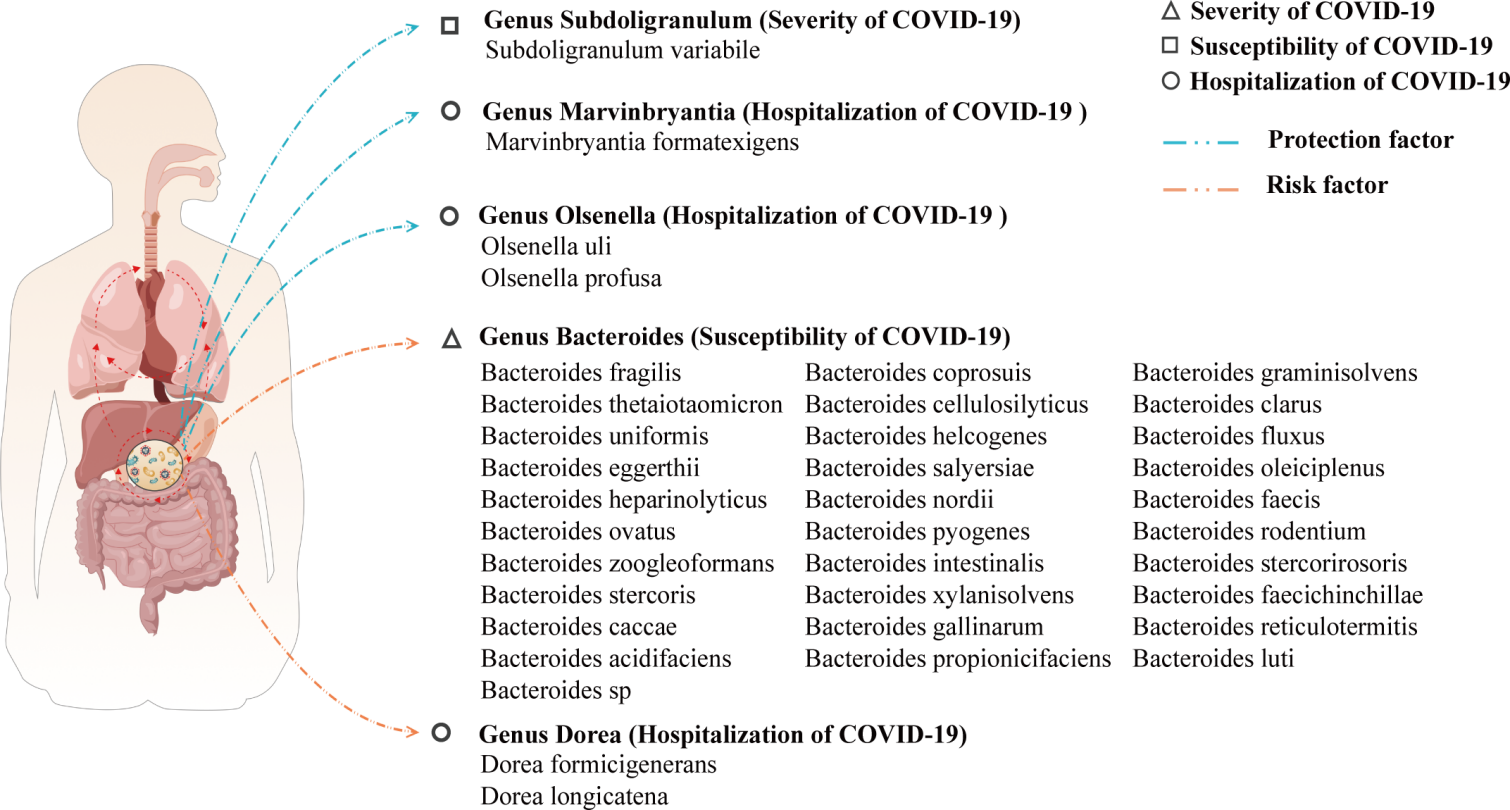
A visualization of the results for predicting species of gut microbiome that may affect COVID-19 based on the gut microbiome genus level.

A search on the UniProtKB and GMrepo websites yielded 37 species of the gut microbiome that may have a causal relationship with COVID-19. Among them, 31 species of the gut microbiome affect COVID-19 susceptibility; 1 species of the gut microbiome affects COVID-19 severity; and 5 species of the gut microbiome affect COVID-19 hospitalization. The likely species of the gut microbiome affecting COVID-19 were predicted by the above steps, and the results are visualized in **Figure 5**.

## 4 Discussion

To our knowledge, this is the first MR study to assess a causal relationship between the gut microbiome and COVID-19 risk. At the locus-wide significance level ( $1\times 10^{-5}$ ), the gut microbiome was found to be strongly associated with COVID-19 disease. Among them, six microbial taxa were causally associated with COVID-19 susceptibility; seven microbial taxa were causally associated with COVID-19 severity risk; five microbial taxa were causally associated with COVID-19 hospitalization. This provides new ideas for the clinical treatment of COVID-19.

Recently, some clinical evidence has shown that respiratory diseases are often accompanied by dysbiosis or changes in the gut microbiome. In studies of the gut microbiome of asthma patients, a link was found between low gut microbial diversity and childhood asthma (26). Asthma has also been linked closely to certain gut microbiome in addition to microbial diversity, such as asthma sufferers’ guts contained more *Clostridium* and *Eggerthella lenta* than healthy controls did (27). A systematic review of 11 studies on respiratory infections and changes in gut microbiome showed that patients with respiratory infections had a 1.45-unit reduction in gut microbiome diversity (Shannon) and a decrease in taxa abundance compared with controls (28). In addition, the antiviral immune response caused by respiratory viral infections such as influenza is associated with changes in the composition and function of gut microbiome (29). Noting that there are alterations in the gut microbiome in respiratory diseases, researchers have sought to prevent and treat respiratory diseases by improving the gut microbiome to modulate the lung immune response (30). A 3-month randomized, double-blind, placebo-controlled human trial in 55 people with asthma demonstrated the adjunct efficacy of *Bifidobacterium lactis Probio* in the treatment of asthma (31). Fecal microbiota transplantation (FMT) experiments in mice with *Streptococcus pneumoniae* infection depleted of gut microbiome detected normalization of lung bacterial counts and tumor necrosis factor-α and interleukin-10 levels, indicating that the gut microbiome plays a role in protecting the host during *Streptococcus pneumoniae* infection (32). A randomized controlled trial of 300 participants found that supplementation with a probiotic formula consisting of *Lactobacillus plantarum* and *Pediococcus acidilactici* improved symptoms in COVID-19 patients (33). Furthermore, a large number of observational studies have found changes in the composition of the gut microbiome in COVID-19 patients compared with healthy controls (34) and that COVID-19 infection increases the number of opportunistic pathogens in the gut and reduces the number of beneficial gut microbiome (35). Through studies of different populations, it was found that the fecal microbiota of COVID-19 patients showed a decrease in bacterial diversity (36) and the richness of the gut microbiome did not return to normal even 6 months after the onset of illness (37). In our MR study, alterations in the gut microbiome of patients with respiratory disease (COVID-19) were also found, suggesting that the gut microbiome we obtained also holds promise for clinical treatment.

Furthermore, understanding the mechanism by which the gut microbiome affects COVID-19 through the gut–lung axis can better provide a target site for the prevention and treatment of COVID-19. The human gut microbiome is made up of more than a trillion bacteria in a dynamic ecosystem that controls our whole physiology and immune system (38). Dysbiosis of the gut microbiome affects not only the immune responses of the gastrointestinal tract but also the immunity of distal organs, such as the lung, which further affects lung health and respiratory conditions (30). Based on previous studies, the gut microbiome can influence the severity of SARS-CoV-2 infection and host immune response through several hypothetical mechanisms: (1) Pro-inflammatory opportunistic pathogens become the dominant strain, which is further recognized by innate lymphocytes and causes a decrease in beneficial metabolites or an increase in pro-inflammatory metabolites (39). (2) Intestinal barrier damage and bacterial displacement can induce an increase in intestinal epithelial reactive oxygen species production as well as increased intestinal permeability, thereby enhancing the intestinal pro-inflammatory response (40). (3) Inflammatory factors reach the lungs through blood circulation, causing the aggregation of inflammatory cells represented by neutrophils and macrophages, and then initiate the inflammatory cascade of the lungs (41, 42). (4) In addition to the peripheral circulation, mesenteric lymph can induce lung damage by transporting enterogenic cytotoxic/inflammatory factors to the pulmonary circulation (41). (5) Opportunistic pathogens and toxins may transfer to the circulatory system, leading to bacteremia and exacerbating systemic inflammation and disease severity (10). In addition, gut microbiome could lead to a decreased expression of angiotensin-converting enzyme 2 (ACE2), an entry receptor for SARS-CoV-2, thereby influencing viral invasion and replication (43, 44). The impact of intestinal ACE2 expression on gut microbiome has been investigated in a number of animal studies. For instance, studies using mouse models revealed that the bacteria *Bacteroides dorei* and *Bifidobacterium longum* decrease colonic ACE2 expression (11, 45). Hirayama et al. identified GATA4, regulated by the gut microbiome, as a transcription factor that regulates ACE2 expression in the gut (46, 47). Therefore, further research into whether the microbial taxa we found also has a similar pathway can further verify the significant role of gut microbiome in COVID-19 and help in the study of targeted drugs.

In this MR study, we obtained that 18 microbial taxa are causally associated with COVID-19. Since the gut microbiome database’s lowest taxonomic level was genus, we predicted 37 species of gut microbiome that affect COVID-19, through the online website. Among them, some gut microbiome and their species can find evidence of association with COVID-19 in previous studies. The proinflammatory effect of *Bacteroides* was validated by GM ecology and correlation analysis against blood inflammatory markers, associated with COVID-19 disease severity (48). In addition, it was discovered that there was a positive correlation between *Bacteroides cellulosilyticus* and the enzymes aspartate transaminase, creatine kinase isoenzymes, and lactate dehydrogenase. This finding suggested that the presence of more gut microorganisms may contribute to severe or critical multiorgan dysfunction (49). Furthermore, there is also evidence of a correlation between *Cyanobacteria*, *Subdolagularum*, and COVID-19 (50, 51). The above results suggest that some of the protective/risky gut microbiome in the results of this MR analysis can be found in previous studies and are likely to become important microbiota for future clinical references. In addition, our newly discovered gut microbiome and predicted species need further improvement in clinical, and *in vivo* experiments to enrich the “gut–lung axis” theories.

The main advantage of this study is that the implementation of the MR method reduces the interference of confounding factors and the reverse causality of results, which may be more persuasive than observational research. However, there are still some limitations: (1) This study only explains the one-way causal relationship between gut microbiome as an exposure factor and COVID-19 as an outcome factor and does not verify the impact of COVID-19 on gut microbiome. (2) The summary-level data of two sample MR was used. Although this method has increased statistical power and obtained interesting results, we cannot adjust important covariates such as diet or drug use. (3) Since most of the participants in GWAS are of European origin, extrapolating the research results to other ethnic groups may be limited. (4) Two GWAS have collected multiple queues, and there may be deviation of overlapping samples. (5) After multiple-testing adjustment, the above association was no longer statistically significant. This MR analysis involved 211 exposures and 3 outcomes, and it is difficult to get meaningful results after multiple validations. Therefore, further experiments are needed to verify the significance of these gut microbiome on COVID-19.

In conclusion, our MR findings suggest a causal effect of the gut microbiome on COVID-19. In addition, genus *Marvinbryantia*, genus *Olsenella*, genus *Ruminococcaceae UCG011*, genus *Subdoligranulum*, and genus *Tyzzerella3* would be potentially beneficial genera for the treatment of COVID-19. This provides new ideas for the development of gut microbiome regulators and new avenues for the treatment of COVID-19 patients.

## Data availability statement

The original contributions presented in the study are included in the article/**Supplementary Material**. Further inquiries can be directed to the corresponding authors.

## Author contributions

Conceptualization, M-MZ, J-HX, YF, and YG. Data curation, J-HX, M-MZ, and YG. Formal analysis, S-HZ and J-NX. Funding acquisition, Y-ZF and YG. Investigation, QZ and X-LS. Methodology, M-MZ and J-HX. Project administration, Y-ZF and YG. Resources, J-HX and S-HZ. Supervision, Y-ZF and YG. Validation, LT and N-XC. Visualization, J-NX and J-HX. Writing of original draft, M-MZ and YF. Writing review and editing, M-MZ, YF, and J-HX. All authors contributed to the article and approved the submitted version.

## Supplementary material

Supplementary Figure 1 Forest plot for causal association between 5 unique protective/risky microbial taxa with COVID-19 hospitalization. **(A, B)** 2 protective microbial taxa for COVID-19 hospitalization; **(C–E)** 3 risky microbial taxa for COVID-19 hospitalization.

Supplementary Figure 2 The plots of the leave-one-out analysis ( $p<1\times 10^{-5}$ ).

## References

[1] Davis HE McCorkell L Vogel JM Topol EJ. Long COVID: major findings, mechanisms and recommendations. Nat Rev Microbiol (2023) 21(3):133–46. doi: 10.1038/s41579-022-00846-2 PMC9839201 36639608

[2] Organization WH. Coronavirus disease (COVID-19) (2023). Available at: https://covid19.who.int/.

[3] Gang J Wang H Xue X Zhang S. Microbiota and COVID-19: Long-term and complex influencing factors. Front Microbiol (2022) 13:963488. doi: 10.3389/fmicb.2022.963488 36033885 PMC9417543

[4] Hadj Hassine I. Covid-19 vaccines and variants of concern: A review. Rev Med Virol (2022) 32(4):e2313. doi: 10.1002/rmv.2313 34755408 PMC8646685

[5] Lin L Jiang X Zhang Z Huang S Zhang Z Fang Z. Gastrointestinal symptoms of 95 cases with SARS-CoV-2 infection. Gut (2020) 69(6):997–1001. doi: 10.1136/gutjnl-2020-321013 32241899

[6] Gilbert JA Blaser MJ Caporaso JG Jansson JK Lynch SV Knight R. Current understanding of the human microbiome. Nat Med (2018) 24(4):392–400. doi: 10.1038/nm.4517 29634682 PMC7043356

[7] Donaldson GP Lee SM Mazmanian SK. Gut biogeography of the bacterial microbiota. Nat Rev Microbiol (2016) 14(1):20–32. doi: 10.1038/nrmicro3552 26499895 PMC4837114

[8] Parrot T Gorin JB Ponzetta A Maleki KT Kammann T Emgård J. MAIT cell activation and dynamics associated with COVID-19 disease severity. Sci Immunol (2020) 5(51):eabe1670. doi: 10.1101/2020.08.27.20182550 32989174 PMC7857393

[9] Legoux F Salou M Lantz O. MAIT cell development and functions: the microbial connection. Immunity (2020) 53(4):710–23. doi: 10.1016/j.immuni.2020.09.009 33053329

[10] Sun Z Song ZG Liu C Tan S Lin S Zhu J. Gut microbiome alterations and gut barrier dysfunction are associated with host immune homeostasis in COVID-19 patients. BMC Med (2022) 20(1):24. doi: 10.1186/s12916-021-02212-0 35045853 PMC8769945

[11] Vatanen T Kostic AD d'Hennezel E Siljander H Franzosa EA Yassour M. Variation in microbiome LPS immunogenicity contributes to autoimmunity in humans. Cell (2016) 165(4):842–53. doi: 10.1016/j.cell.2016.04.007 PMC4950857 27133167

[12] Gaibani P D'Amico F Bartoletti M Lombardo D Rampelli S Fornaro G. The gut microbiota of critically ill patients with COVID-19. Front Cell Infect Microbiol (2021) 11:670424. doi: 10.3389/fcimb.2021.670424 34268136 PMC8276076

[13] Ren Z Wang H Cui G Lu H Wang L Luo H. Alterations in the human oral and gut microbiomes and lipidomics in COVID-19. Gut (2021) 70(7):1253–65. doi: 10.1136/gutjnl-2020-323826 PMC8042598 33789966

[14] Arsenault BJ. From the garden to the clinic: how Mendelian randomization is shaping up atherosclerotic cardiovascular disease prevention strategies. Eur Heart J (2022) 43(42):4447–9. doi: 10.1093/eurheartj/ehac394 35869924

[15] Emdin CA Khera AV Kathiresan S. Mendelian randomization. Jama (2017) 318(19):1925–6. doi: 10.1001/jama.2017.17219 29164242

[16] Bowden J Holmes MV. Meta-analysis and Mendelian randomization: A review. Res Synth Methods (2019) 10(4):486–96. doi: 10.1002/jrsm.1346 PMC6973275 30861319

[17] Kurilshikov A Medina-Gomez C Bacigalupe R Radjabzadeh D Wang J Demirkan A. Large-scale association analyses identify host factors influencing human gut microbiome composition. Nat Genet (2021) 53(2):156–65. doi: 10.1038/s41588-020-00763-1 PMC8515199 33462485

[18] Initiative C-HG. The COVID-19 Host Genetics Initiative, a global initiative to elucidate the role of host genetic factors in susceptibility and severity of the SARS-CoV-2 virus pandemic. Eur J Hum Genet (2020) 28(6):715–8. doi: 10.1002/jrsm.1346 PMC7220587 32404885

[19] Burgess S Butterworth A Thompson SG. Mendelian randomization analysis with multiple genetic variants using summarized data. Genet Epidemiol (2013) 37(7):658–65. doi: 10.1002/gepi.21758 PMC4377079 24114802

[20] Bowden J Davey Smith G Burgess S. Mendelian randomization with invalid instruments: effect estimation and bias detection through Egger regression. Int J Epidemiol (2015) 44(2):512–25. doi: 10.1093/ije/dyv080 PMC4469799 26050253

[21] Bowden J Davey Smith G Haycock PC Burgess S. Consistent estimation in mendelian randomization with some invalid instruments using a weighted median estimator. Genet Epidemiol (2016) 40(4):304–14. doi: 10.1002/gepi.21965 PMC4849733 27061298

[22] Verbanck M Chen CY Neale B Do R. Detection of widespread horizontal pleiotropy in causal relationships inferred from Mendelian randomization between complex traits and diseases. Nat Genet (2018) 50(5):693–8. doi: 10.1038/s41588-018-0099-7 PMC6083837 29686387

[23] Burgess S Thompson SG. Interpreting findings from Mendelian randomization using the MR-Egger method. Eur J Epidemiol (2017) 32(5):377–89. doi: 10.1007/s10654-017-0255-x PMC5506233 28527048

[24] UniProt: the universal protein knowledgebase in 2023. Nucleic Acids Res (2023) 51(D1):D523–31. doi: 10.1093/nar/gkac1052 PMC9825514 36408920

[25] Dai D Zhu J Sun C Li M Liu J Wu S. GMrepo v2: a curated human gut microbiome database with special focus on disease markers and cross-dataset comparison. Nucleic Acids Res (2022) 50(D1):D777–d84. doi: 10.1093/nar/gkab1019 PMC8728112 34788838

[26] Abrahamsson TR Jakobsson HE Andersson AF Björkstén B Engstrand L Jenmalm MC. Low gut microbiota diversity in early infancy precedes asthma at school age. Clin Exp Allergy (2014) 44(6):842–50. doi: 10.1111/cea.12253 24330256

[27] Wang Q Li F Liang B Liang Y Chen S Mo X. A metagenome-wide association study of gut microbiota in asthma in UK adults. BMC Microbiol (2018) 18(1):114. doi: 10.1186/s12866-018-1257-x 30208875 PMC6134768

[28] Woodall CA McGeoch LJ Hay AD Hammond A. Respiratory tract infections and gut microbiome modifications: A systematic review. PloS One (2022) 17(1):e0262057. doi: 10.1371/journal.pone.0262057 35025938 PMC8757905

[29] Hanada S Pirzadeh M Carver KY Deng JC. Respiratory viral infection-induced microbiome alterations and secondary bacterial pneumonia. Front Immunol (2018) 9:2640. doi: 10.3389/fimmu.2018.02640 30505304 PMC6250824

[30] Chunxi L Haiyue L Yanxia L Jianbing P Jin S. The gut microbiota and respiratory diseases: new evidence. J Immunol Res (2020) 2020:2340670. doi: 10.1155/2020/2340670 32802893 PMC7415116

[31] Liu A Ma T Xu N Jin H Zhao F Kwok LY. Adjunctive probiotics alleviates asthmatic symptoms via modulating the gut microbiome and serum metabolome. Microbiol Spectr (2021) 9(2):e0085921. doi: 10.1128/Spectrum.00859-21 34612663 PMC8510161

[32] Schuijt TJ Lankelma JM Scicluna BP de Sousa e Melo F Roelofs JJ de Boer JD. The gut microbiota plays a protective role in the host defence against pneumococcal pneumonia. Gut (2016) 65(4):575–83. doi: 10.1136/gutjnl-2015-309728 PMC4819612 26511795

[33] Gutiérrez-Castrellón P Gandara-Martí T Abreu YAAT Nieto-Rufino CD López-Orduña E Jiménez-Escobar I. Probiotic improves symptomatic and viral clearance in Covid19 outpatients: a randomized, quadruple-blinded, placebo-controlled trial. Gut Microbes (2022) 14(1):2018899. doi: 10.1080/19490976.2021.2018899 35014600 PMC8757475

[34] Zhang F Lau RI Liu Q Su Q Chan FKL Ng SC. Gut microbiota in COVID-19: key microbial changes, potential mechanisms and clinical applications. Nat Rev Gastroenterol Hepatol (2022) 20(5):323–37. doi: 10.1038/s41575-022-00698-4 PMC9589856 36271144

[35] Zuo T Zhan H Zhang F Liu Q Tso EYK Lui GCY. Alterations in fecal fungal microbiome of patients with COVID-19 during time of hospitalization until discharge. Gastroenterology (2020) 159(4):1302–10.e5. doi: 10.1053/j.gastro.2020.06.048 32598884 PMC7318920

[36] Xu R Lu R Zhang T Wu Q Cai W Han X. Temporal association between human upper respiratory and gut bacterial microbiomes during the course of COVID-19 in adults. Commun Biol (2021) 4(1):240. doi: 10.1038/s42003-021-01796-w 33603076 PMC7893062

[37] Chen Y Gu S Chen Y Lu H Shi D Guo J. Six-month follow-up of gut microbiota richness in patients with COVID-19. Gut (2022) 71(1):222–5. doi: 10.1136/gutjnl-2021-324090 PMC8666823 33833065

[38] de Oliveira GLV Oliveira CNS Pinzan CF de Salis LVV Cardoso CRB. Microbiota modulation of the gut-lung axis in COVID-19. Front Immunol (2021) 12:635471. doi: 10.3389/fimmu.2021.635471 33717181 PMC7945592

[39] Constantinides MG. Interactions between the microbiota and innate and innate-like lymphocytes. J Leukoc Biol (2018) 103(3):409–19. doi: 10.1002/JLB.3RI0917-378R 29345366

[40] Uzzan M Corcos O Martin JC Treton X Bouhnik Y. Why is SARS-CoV-2 infection more severe in obese men? The gut lymphatics - Lung axis hypothesis. Med Hypotheses (2020) 144:110023. doi: 10.1016/j.mehy.2020.110023 32593832 PMC7308746

[41] Ma Y Yang X Chatterjee V Wu MH Yuan SY. The gut-lung axis in systemic inflammation. Role of mesenteric lymph as a conduit. Am J Respir Cell Mol Biol (2021) 64(1):19–28. doi: 10.1165/rcmb.2020-0196TR 32877613 PMC7781004

[42] Young RP Hopkins RJ Marsland B. The gut-liver-lung axis. Modulation of the innate immune response and its possible role in chronic obstructive pulmonary disease. Am J Respir Cell Mol Biol (2016) 54(2):161–9. doi: 10.1165/rcmb.2015-0250PS 26473323

[43] Zhang H Li HB Lyu JR Lei XM Li W Wu G. Specific ACE2 expression in small intestinal enterocytes may cause gastrointestinal symptoms and injury after 2019-nCoV infection. Int J Infect Dis (2020) 96:19–24. doi: 10.1016/j.ijid.2020.04.027 32311451 PMC7165079

[44] Viana SD Nunes S Reis F. ACE2 imbalance as a key player for the poor outcomes in COVID-19 patients with age-related comorbidities - Role of gut microbiota dysbiosis. Ageing Res Rev (2020) 62:101123. doi: 10.1016/j.arr.2020.101123 32683039 PMC7365123

[45] Geva-Zatorsky N Sefik E Kua L Pasman L Tan TG Ortiz-Lopez A. Mining the human gut microbiota for immunomodulatory organisms. Cell (2017) 168(5):928–43.e11. doi: 10.1016/j.cell.2017.01.022 28215708 PMC7774263

[46] Hirayama M Nishiwaki H Hamaguchi T Ito M Ueyama J Maeda T. Intestinal Collinsella may mitigate infection and exacerbation of COVID-19 by producing ursodeoxycholate. PloS One (2021) 16(11):e0260451. doi: 10.1371/journal.pone.0260451 34813629 PMC8610263

[47] Liu Y Zhang H Tang X Jiang X Yan X Liu X. Distinct metagenomic signatures in the SARS-coV-2 infection. Front Cell Infect Microbiol (2021) 11:706970. doi: 10.3389/fcimb.2021.706970 34926314 PMC8674698

[48] ROmani L Del Chierico F Macari G Pane S Ristori MV Guarrasi V. The relationship between pediatric gut microbiota and SARS-coV-2 infection. Front Cell Infect Microbiol (2022) 12:908492. doi: 10.3389/fcimb.2022.908492 35873161 PMC9304937

[49] Xu X Zhang W Guo M Xiao C Fu Z Yu S. Integrated analysis of gut microbiome and host immune responses in COVID-19. Front Med (2022) 16(2):263–75. doi: 10.1007/s11684-022-0921-6 PMC8902486 35258762

[50] Barre A Van Damme EJM Simplicien M Le Poder S Klonjkowski B Benoist H. Man-specific lectins from plants, fungi, algae and cyanobacteria, as potential blockers for SARS-CoV, MERS-CoV and SARS-CoV-2 (COVID-19) coronaviruses: biomedical perspectives. Cells (2021) 10(7):1619. doi: 10.3390/cells10071619 34203435 PMC8305077

[51] Vestad B Ueland T Lerum TV Dahl TB Holm K Barratt-Due A. Respiratory dysfunction three months after severe COVID-19 is associated with gut microbiota alterations. J Intern Med (2022) 291(6):801–12. doi: 10.1111/joim.13458 PMC9115297 35212063

