# Supplementary figures and images for: Causal effects of the gut microbiome on COVID-19 susceptibility and severity: a two-sample Mendelian randomization study
Source: Front Immunol. 2023 Sep 1;14:1173974. doi: 10.3389/fimmu.2023.1173974 (PMC10502427; doi:10.3389/fimmu.2023.1173974)

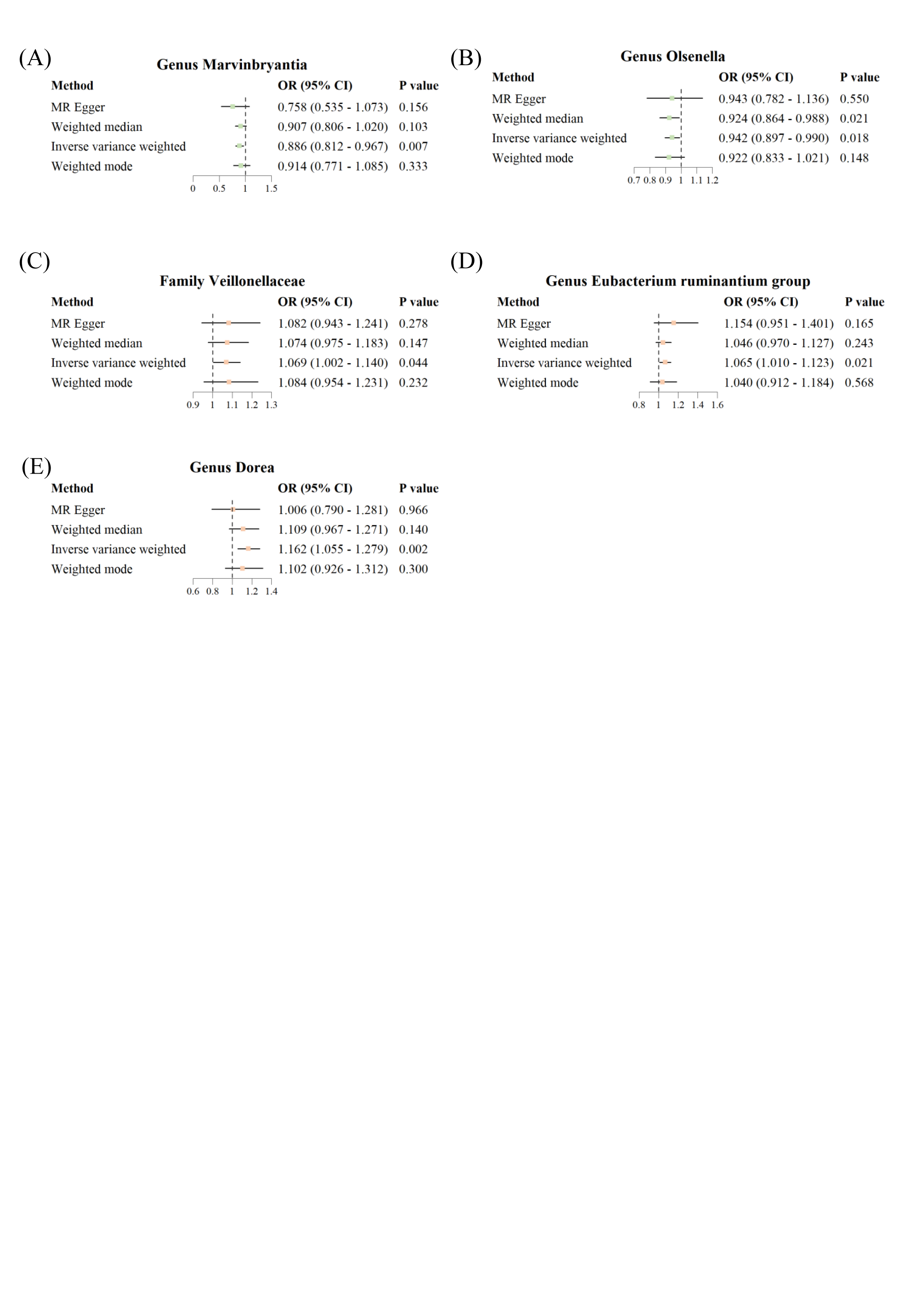

Supplement: Supplementary Figure 1 — Forest plot for causal association between 5 unique protective/risky microbial taxa with COVID-19 hospitalization. (A, B) 2 protective microbial taxa for COVID-19 hospitalization; (C–E) 3 risky microbial taxa for COVID-19 hospitalization. [file Image_1.tif]

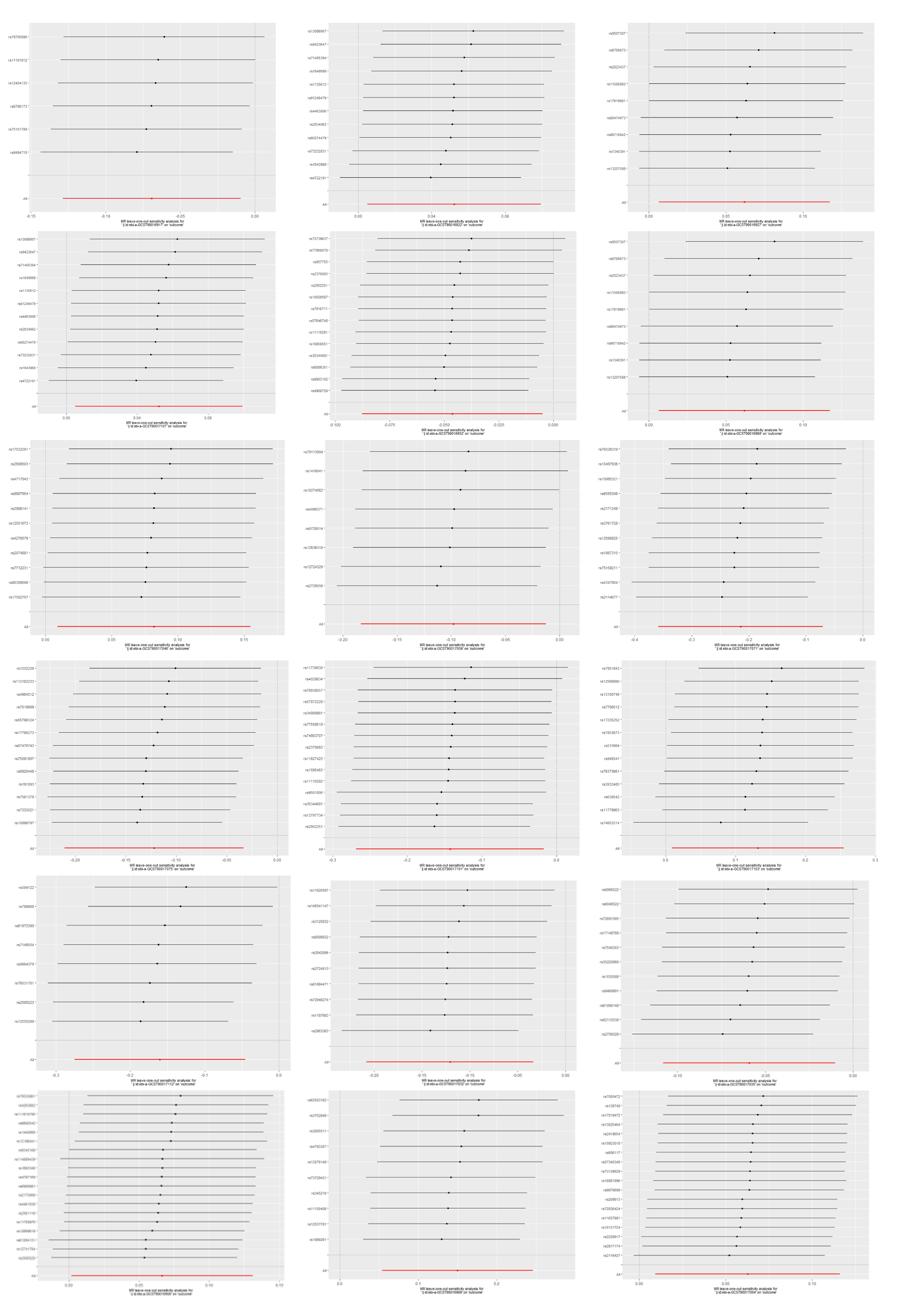

Supplement: Supplementary Figure 2 — The plots of the leave-one-out analysis ( p<1×10^−5^ ). [file Image_2.tif]
