# Supplementary material for: Causal effects of the gut microbiome on COVID-19 susceptibility and severity: a two-sample Mendelian randomization study
Source: Front Immunol. 2023 Sep 1;14:1173974. doi: 10.3389/fimmu.2023.1173974 (PMC10502427; doi:10.3389/fimmu.2023.1173974)
Supplement: Supplementary file 3 [file Table_1.docx]

**Supplementary Table 1** **MR results of causal links between gut microbiome and COVID-19 susceptibility risk (P < 1 × 10-5)**

| Outcome |  | Nsnp | Method | Beta | SE | P value | OR(95%CI) | Factor |
| --- | --- | --- | --- | --- | --- | --- | --- | --- |
| Class | Gammaproteobacteria | 6 | MR Egger | -0.129 | 0.102 | 0.275 | 0.879(0.720-1.074) | protect |
|  |  |  | Weighted median | -0.078 | 0.036 | 0.031 | 0.925(0.862-0.993) |  |
|  |  |  | Inverse variance weighted | -0.069 | 0.030 | 0.023 | 0.933(0.879-0.991) |  |
|  |  |  | Weighted mode | -0.085 | 0.049 | 0.141 | 0.919(0.835-1.010) |  |
|  | Negativicutes | 12 | MR Egger | 0.033 | 0.080 | 0.690 | 1.033(0.883-1.209) | risk |
|  |  |  | Weighted median | 0.061 | 0.034 | 0.074 | 1.063(0.994-1.136) |  |
|  |  |  | Inverse variance weighted | 0.052 | 0.024 | 0.030 | 1.054(1.005-1.105) |  |
|  |  |  | Weighted mode | 0.067 | 0.056 | 0.256 | 1.070(0.958-1.194) |  |
| Family | Bacteroidaceae | 9 | MR Egger | 0.017 | 0.159 | 0.919 | 1.017(0.745-1.388) | risk |
|  |  |  | Weighted median | 0.052 | 0.038 | 0.166 | 1.054(0.979-1.134) |  |
|  |  |  | Inverse variance weighted | 0.062 | 0.028 | 0.028 | 1.064(1.007-1.125) |  |
|  |  |  | Weighted mode | 0.051 | 0.053 | 0.370 | 1.052(0.948-1.167) |  |
|  | Streptococcaceae | 14 | MR Egger | -0.165 | 0.086 | 0.079 | 0.848(0.717-1.003) | protect |
|  |  |  | Weighted median | -0.045 | 0.028 | 0.107 | 0.956(0.905-1.010) |  |
|  |  |  | Inverse variance weighted | -0.046 | 0.021 | 0.029 | 0.955(0.916-0.995) |  |
|  |  |  | Weighted mode | -0.043 | 0.046 | 0.359 | 0.958(0.876-1.047) |  |
| Genus | Bacteroides | 9 | MR Egger | 0.017 | 0.159 | 0.919 | 1.017(0.745-1.388) | risk |
|  |  |  | Weighted median | 0.052 | 0.038 | 0.172 | 1.054(0.978-1.135) |  |
|  |  |  | Inverse variance weighted | 0.062 | 0.028 | 0.028 | 1.064(1.007-1.125) |  |
|  |  |  | Weighted mode | 0.051 | 0.056 | 0.391 | 1.052(0.943-1.173) |  |
| Order | Selenomonadales | 12 | MR Egger | 0.033 | 0.080 | 0.690 | 1.033(0.883-1.209) | risk |
|  |  |  | Weighted median | 0.061 | 0.033 | 0.067 | 1.063(0.996-1.134) |  |
|  |  |  | Inverse variance weighted | 0.052 | 0.024 | 0.030 | 1.054(1.005-1.105) |  |
|  |  |  | Weighted mode | 0.067 | 0.050 | 0.203 | 1.070(0.970-1.179) |  |

**Supplementary Table 2** **MR results of causal links between gut microbiome and COVID-19 severity risk (P < 1 × 1 0-5)**

| Outcome |  | Nsnp | Method | Beta | SE | P value | OR(95%CI) | Factor |
| --- | --- | --- | --- | --- | --- | --- | --- | --- |
| Genus | Rikenellaceae RC9 gut group | 11 | MR Egger | 0.058 | 0.229 | 0.805 | 1.060(0.677-1.660) | risk |
|  |  |  | Weighted median | 0.082 | 0.048 | 0.085 | 1.086(0.989-1.192) |  |
|  |  |  | Inverse variance weighted | 0.082 | 0.037 | 0.028 | 1.085(1.009-1.167) |  |
|  |  |  | Weighted mode | 0.092 | 0.074 | 0.242 | 1.096(0.948-1.268) |  |
|  | Ruminococcaceae UCG011 | 8 | MR Egger | -0.254 | 0.224 | 0.300 | 0.776(0.500-1.203) | protect |
|  |  |  | Weighted median | -0.098 | 0.053 | 0.064 | 0.906(0.817-1.006) |  |
|  |  |  | Inverse variance weighted | -0.098 | 0.044 | 0.025 | 0.907(0.832-0.988) |  |
|  |  |  | Weighted mode | -0.128 | 0.086 | 0.180 | 0.880(0.743-1.041) |  |
|  | Subdoligranulum | 11 | MR Egger | -0.118 | 0.179 | 0.528 | 0.889(0.625-1.264) | protect |
|  |  |  | Weighted median | -0.138 | 0.100 | 0.168 | 0.871(0.716-1.060) |  |
|  |  |  | Inverse variance weighted | -0.214 | 0.073 | 0.004 | 0.807(0.699-0.932) |  |
|  |  |  | Weighted mode | -0.112 | 0.157 | 0.494 | 0.894(0.657-1.217) |  |
|  | Tyzzerella3 | 13 | MR Egger | -0.040 | 0.279 | 0.890 | 0.961(0.556-1.662) | protect |
|  |  |  | Weighted median | -0.124 | 0.060 | 0.038 | 0.884(0.786-0.993) |  |
|  |  |  | Inverse variance weighted | -0.122 | 0.045 | 0.007 | 0.885(0.810-0.967) |  |
|  |  |  | Weighted mode | -0.132 | 0.097 | 0.195 | 0.876(0.725-1.058) |  |
| Order | Lactobacillales | 15 | MR Egger | -0.129 | 0.161 | 0.436 | 0.879(0.641-1.204) | protect |
|  |  |  | Weighted median | -0.134 | 0.092 | 0.147 | 0.875(0.730-1.048) |  |
|  |  |  | Inverse variance weighted | -0.143 | 0.064 | 0.026 | 0.867(0.764-0.983) |  |
|  |  |  | Weighted mode | -0.138 | 0.127 | 0.295 | 0.871(0.680-1.117) |  |
|  | Mollicutes RF9 | 13 | MR Egger | 0.330 | 0.187 | 0.105 | 1.391(0.964-2.008) | risk |
|  |  |  | Weighted median | 0.092 | 0.080 | 0.250 | 1.096(0.938-1.282) |  |
|  |  |  | Inverse variance weighted | 0.132 | 0.063 | 0.035 | 1.141(1.009-1.291) |  |
|  |  |  | Weighted mode | 0.054 | 0.150 | 0.724 | 1.056(0.787-1.417) |  |
| Phylum | Cyanobacteria | 8 | MR Egger | -0.242 | 0.224 | 0.321 | 0.785(0.506-1.218) | protect |
|  |  |  | Weighted median | -0.134 | 0.080 | 0.097 | 0.875(0.747-1.024) |  |
|  |  |  | Inverse variance weighted | -0.160 | 0.059 | 0.006 | 0.852(0.760-0.955) |  |
|  |  |  | Weighted mode | -0.137 | 0.113 | 0.266 | 0.872(0.698-1.089) |  |

**Supplementary Table 3** **MR results of causal links between gut microbiome and COVID-19 hospitalization risk (P < 1 × 1 0-5)**

| Outcome |  | Nsnp | Method | Beta | SE | P value | OR(95%CI) | Factor |
| --- | --- | --- | --- | --- | --- | --- | --- | --- |
| Genus | Marvinbryantia | 10 | MR Egger | -0.277 | 0.177 | 0.156 | 0.758(0.535-1.073) | protect |
|  |  |  | Weighted median | -0.098 | 0.060 | 0.103 | 0.907(0.806-1.020) |  |
|  |  |  | Inverse variance weighted | -0.121 | 0.045 | 0.007 | 0.886(0.812-0.967) |  |
|  |  |  | Weighted mode | -0.089 | 0.087 | 0.333 | 0.914(0.771-1.085) |  |
|  | Olsenella | 11 | MR Egger | -0.059 | 0.095 | 0.550 | 0.943(0.782-1.136) | protect |
|  |  |  | Weighted median | -0.079 | 0.034 | 0.021 | 0.924(0.864-0.988) |  |
|  |  |  | Inverse variance weighted | -0.059 | 0.025 | 0.018 | 0.942(0.897-0.990) |  |
|  |  |  | Weighted mode | -0.081 | 0.052 | 0.148 | 0.922(0.833-1.021) |  |
|  | Dorea | 10 | MR Egger | 0.006 | 0.123 | 0.966 | 1.006(0.790-1.281) | risk |
|  |  |  | Weighted median | 0.103 | 0.070 | 0.140 | 1.109(0.967-1.271) |  |
|  |  |  | Inverse variance weighted | 0.150 | 0.049 | 0.002 | 1.162(1.055-1.279) |  |
|  |  |  | Weighted mode | 0.097 | 0.089 | 0.300 | 1.102(0.926-1.312) |  |
|  | Eubacterium ruminantium group | 18 | MR Egger | 0.144 | 0.099 | 0.165 | 1.154(0.951-1.401) | risk |
|  |  |  | Weighted median | 0.045 | 0.038 | 0.243 | 1.046(0.970-1.127) |  |
|  |  |  | Inverse variance weighted | 0.063 | 0.027 | 0.021 | 1.065(1.010-1.123) |  |
|  |  |  | Weighted mode | 0.039 | 0.067 | 0.568 | 1.040(0.912-1.184) |  |
| Family | Veillonellaceae | 19 | MR Egger | 0.078 | 0.070 | 0.278 | 1.082(0.943-1.241) | risk |
|  |  |  | Weighted median | 0.071 | 0.049 | 0.147 | 1.074(0.975-1.183) |  |
|  |  |  | Inverse variance weighted | 0.066 | 0.033 | 0.044 | 1.069(1.002-1.140) |  |
|  |  |  | Weighted mode | 0.080 | 0.065 | 0.232 | 1.084(0.954-1.231) |  |
